# Network pharmacology-based prediction of the multi-target capabilities of the compounds in Taohong Siwu decoction, and their application in osteoarthritis

**DOI:** 10.3892/etm.2013.1106

**Published:** 2013-05-09

**Authors:** CHUN-SONG ZHENG, XIAO-JIE XU, HONG-ZHI YE, GUANG-WEN WU, XI-HAI LI, HUI-FENG XU, XIAN-XIANG LIU

**Affiliations:** 1 Fujian Academy of Integrative Medicine, Fujian University of Traditional Chinese Medicine, Fuzhou, Fujian 350122;; 2 Fujian Key Laboratory of Integrative Medicine on Geriatrics, Fujian University of Traditional Chinese Medicine, Fuzhou, Fujian 350122;; 3 College of Chemistry and Molecular Engineering, Peking University, Beijing 100871, P.R. China

**Keywords:** Taohong Siwu decoction, osteoarthritis, multi-target, network pharmacology

## Abstract

Taohong Siwu decoction (THSWD), a formulation prescribed in traditional Chinese medicine (TCM), has been widely used in the treatment of osteoarthritis (OA). TCM has the potential to prevent diseases, such as OA, in an integrative and holistic manner. However, the system-level characterization of the drug-target interactions of THSWD has not been elucidated. In the present study, we constructed a novel modeling system, by integrating chemical space, virtual screening and network pharmacology, to investigate the molecular mechanism of action of THSWD. The chemical distribution of the ligand database and the potential compound prediction demonstrated that THSWD, as a natural combinatorial chemical library, comprises abundant drug-like and lead-like compounds that may act as potential inhibitors for a number of important target proteins associated with OA. Moreover, the results of the ‘compound-target network’ analysis demonstrated that 19 compounds within THSWD were correlated with more than one target, whilst the maximum degree of correlation for the compounds was seven. Furthermore, the ‘target-disease network’ indicated that THSWD may potentially be effective against 69 diseases. These results may aid in the understanding of the use of THSWD as a multi-target therapy in OA. Moreover, they may be useful in establishing other pharmacological effects that may be brought about by THSWD. The *in silico* method used in this study has the potential to advance the understanding of the molecular mechanisms of TCM.

## Introduction

Osteoarthritis (OA) is a disease that is induced through several complex mechanisms, including the progressive erosion of articular cartilage, proteoglycan (PG) degradation and the disruption of the collagen network. It is the most common form of arthritis, and is a major cause of morbidity, limitations in activity, physical disability, excessive health care utilization and a reduced health-related quality of life, particularly in individuals aged ≥45 years (1). Treatments for OA are widely perceived as an unmet medical requirement. Until recently, there has been no specific remedy for the disease, despite the availability of supplements, such as chondroitin sulfate, and the wide range of effective analgesics and non-steroidal anti-inflammatory drugs (NSAIDs) that are used as treatments for OA. However, the pharmacological management of OA has targeted the symptoms of the disease, rather than the underlying causes (2,3). Thus, there have been efforts to develop drugs that reverse, retard or stabilize the underlying pathological changes in OA, and thereby provide a long-term symptomatic relief.

At present, efforts are being focused on the discovery of drugs that affect multiple targets and levels of the disease network. A diverse range of studies have been performed in the emerging field of multi-target drug design (4–7). In addition, several studies have demonstrated that an improved method of treating OA may be to act on several targets simultaneously (6,8). It has also been indicated that the substances used in traditional Chinese medicine (TCM) possess diverse biological activities, and their composition and pharmacodynamic action are improved when administered in conjunction, as opposed to individually (9). Therefore, the establishment of a comprehensive method that elucidates the multi-target capabilities of TCM is required for the development of new drugs to treat OA.

Taohong Siwu decoction (THSWD) is a TCM that was documented in ‘YiZong JinJian’ *(*compiled by Wu Qian in the Qing Dynasty), and has been prescribed to treat OA in China. It has been demonstrated that THSWD effectively inhibits the expression of matrix metalloproteinase (MMP)-1 in the articular cartilage of rats with knee osteoarthritis (KOA), modeled by Hulth’s method, and reduces the levels of interleukin (IL)-l and IL-6 in the localized tissues of the KOA (10,11). However, the underlying mechanisms of THSWD in the management of OA are poorly understood. With the progress that has been made in systems biology and bionetworks, network pharmacology has been used to study the functions of herbal formulations from a proteome or systematic level (12). Therefore, the aim of the present study was to elucidate the multi-target capabilities of the compounds in THSWD, based on the established methods of network pharmacology (13,14). In addition, the study aimed to promote the understanding of the efficiency of THSWD, with regard to its application in OA, and to facilitate its modernization and global use.

## Materials and methods

### Construction and preparation of the compound ligand data-base

THSWD consists of six species of medicinal herbs: *Radix Rehmanniae Preparata* (Shudihuang), *Angelica sinensis* (Danggui), *Radix Paeoniae Alba* (Baishao), *Ligusticum chuanxiong* (Chuanxiong), *Prunus persica* (Taoren) and *Carthamus annuum* (Honghua). The compounds identified in the medicinal herbs of THSWD were determined from the Chinese Herbal Drug Database and the Handbook of the Chemical Constituents in Chinese Herb Original Plants (15,16). Having excluded any duplicates, the total number of compounds was 206. The structures of the compounds were drawn using ISIS Draw, Version 2.5 (MDL Information Systems, Inc., San Leandro, CA, USA), and further optimized using Discovery Studio 2.0 (DS 2.0; Accelrys, Inc., San Diego, CA, USA), with a Merck molecular force field (MMFF).

The molecular descriptors of the ligand database were calculated in the quantitative structure-activity relationship (QSAR) module of DS 2.0 (Accelrys, Inc.), whilst the chemical space of the ligand database was constructed using 150 diversity descriptors, including 1D, 2D and 3D molecular descriptors. Principal component analysis (PCA) was then performed to map the chemical distribution of the ligands in chemical space.

### Virtual docking screening

To highlight the THSWD components that were particularly likely to be active in the OA disease system, a docking protocol was performed, in order to identify any interactions with the common OA target enzymes. This was completed using DS 2.0 LigandFit (Accelrys, Inc.) (7).

The 3D crystal structures of the 15 protein targets associated with OA, as determined by the Therapeutic Targets Database (TTD; Bioinformatics and Drug Design group, National University of Singapore) and other literature sources (17–20), were obtained from the Research Collabatory for Structural Bioinformatics (RCSB) Protein Data Bank (PDB; Table I), and uploaded into DS 2.0 (Accelrys, Inc.). The crystallographic water was removed, and hydrogen atoms were added. The inhibitor from the PDB file was used to define the active site, and the 206 compounds identified in THSWD were docked into the protein models. The ligand position and orientation were evaluated using DockScores, on the basis of the most favorable energy interactions between the ligand conformations and receptor proteins, as described previously (21). The 206 docked structures were thus sorted according to their DockScores.

### Network construction and analysis

Three networks were constructed for the purpose of the study. To create a candidate compound-candidate target (cC-cT) network, 242 drug/drug-like compounds associated with the 15 previously mentioned protein targets, were obtained from the TTD (17). Known compounds and their targets were used to generate a bipartite graph of compound-protein interactions, in which a compound and a protein were connected to each other if the protein was an action target of the compound. This gave rise to the cC-cT network. To create a potential compound-potential target (pC-pT) network, the top five of the DockScore-sorted THSWD compounds were selected as potential compounds (18). The pC-pT network was established by connecting the potential compounds to any corresponding potential targets that were associated with OA. The target-disease (T-D) network was constructed by connecting the 15 previously mentioned proteins to any associated diseases, as established from the TTD. Cytoscape 2.8.3 (Cytoscape Consortium; http://www.cytoscape.org/) was used in the construction of all the networks, as described previously (22). The compounds, targets and diseases were represented as nodes in the networks. The edges between the nodes represented intermolecular interactions. Data were analyzed using Cytoscape plugins.

## Results

### Chemical distribution of the ligand database

**Figure 1.**
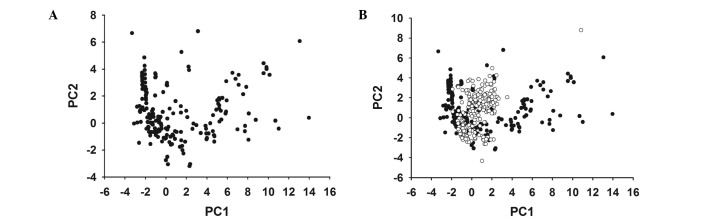
Global property map of the chemical space of compounds. The black circles represent the compounds retrieved from Taohong Siwu decoction, while the white circles represent the compounds retrieved from the Therapeutic Targets Database (TTD; Bioinformatics and Drug Design group, National University of Singapore). PC1, first principal component; PC2,second principal component.

The global property map of the chemical space of the compounds in THSWD is depicted in Fig. 1A, which demonstrates that the compounds possess a broad diversity in chemical space. In addition, several key molecular descriptors were statistically analyzed, and are listed in Table II. These results indicated that the majority of the THSWD compounds had desired drug-like properties, according to Lipinski’s rule of five. To further confirm the drug-like properties of the THSWD compounds, the chemical space distribution of the known drug/drug-like compounds associated with the OA targets, as established from the TTD, were also charted. It was revealed that there were marked overlaps between these two molecular datasets in chemical space (Fig. 1B), which indicated that THSWD contained drug-like and lead-like compounds.

### Potential compound prediction

The potential compound prediction was performed for the 15 proteins associated with OA using virtual screening. Docking revealed that the total number of potential compounds that exhibited desired interactions with the proteins was 38. Among these 38 compounds, 19 demonstrated potential biological activity with more than one target protein. These results indicated that THSWD contained a number of active compounds, which had the potential to lead to the broad-spectrum inhibition of a number of important target proteins.

### C-T network construction and analysis results

**Figure 2.**
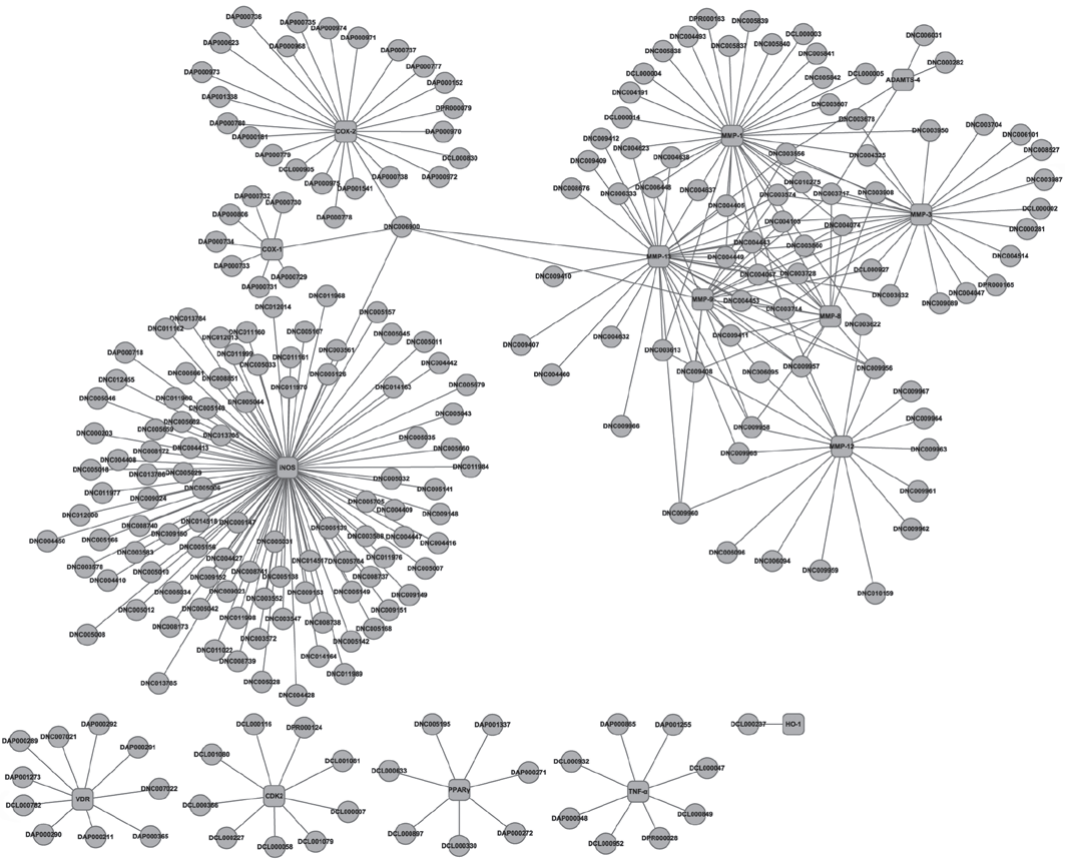
Candidate compound-candidate target (cC-cT) network. The circles and rounded rectangles represent the candidate compounds and target proteins, respectively.

**Figure 3.**
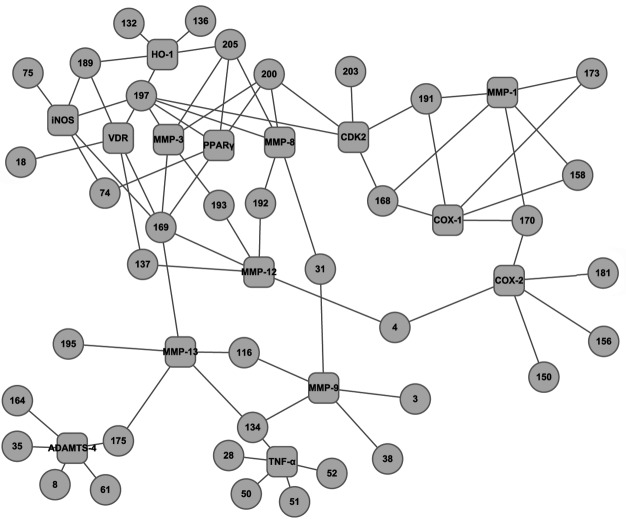
Potential compound-potential target (pC-pT) network. The circles and rounded rectangles represent the potential compounds and target proteins, respectively.

**Figure 4.**
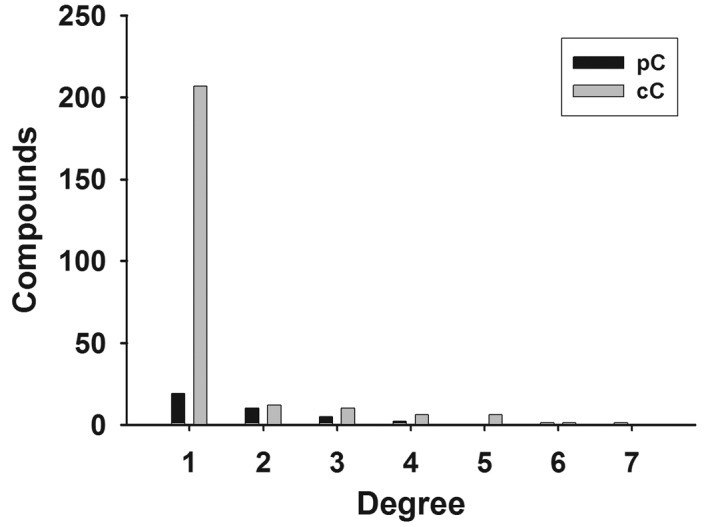
Distribution of the number of targets associated with each compound. pC, potential compound; cC, candidate compound.

The cC-cT and pC-pT networks are demonstrated in Figs. 2 and 3, respectively. A histogram of the distribution of the number of targets correlated with each compound, and corresponding with the cC-cT and pC-pT networks, is demonstrated in Fig. 4. The simple parameters of the cC-cT and pC-pT networks are exhibited in Table III. These results indicated that the majority of the candidate and potential compounds interacted with only one target protein; however, certain compounds were identified to be modulators of multiple targets.

### T-D network construction and analysis results

**Figure 5.**
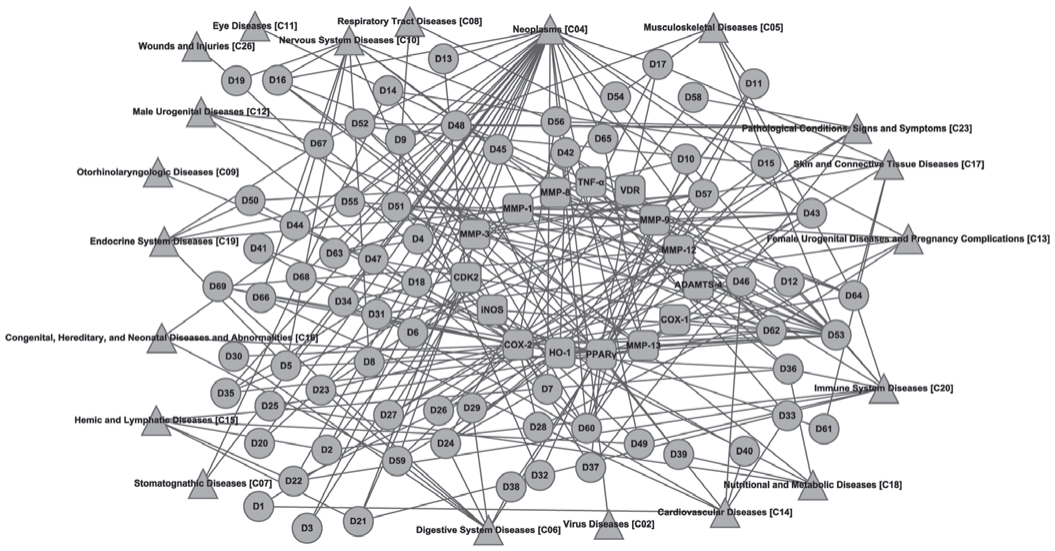
Target-disease network of 15 potential targets (rounded rectangles) connected to 69 diseases (circles), which were classified into 20 groups (triangles) according to the US National Library of Medicine’s Medical Subject Headings (http://www.nlm.nih.gov/mesh).

A total of 15 validated potential targets and the 69 diseases that were associated with them, as established from the TTD website, were used to construct the T-D network (Table IV and Fig. 5). According to the US National National Library’s Medical Subject Headings (http://www.nlm.nih.gov/mesh), the 69 diseases were classified into 20 groups. For example, OA, osteoporosis and rheumatoid arthritis were grouped as musculoskeletal diseases, while heart failure, myocardial infarction and ischemia reperfusion injuries were classified as cardiovascular diseases. This suggests that THSWD may demonstrate efficacy in targeting not only musculoskeletal diseases, but also neoplasms, nervous system diseases and cardiovascular diseases, amongst others.

## Discussion

The chemical space of the compounds in THSWD was defined by calculating a given set of descriptors for each molecule, and by using these values as coordinates in the multidimensional space. PCA was performed to map the multiple descriptor values into a 2D plane. This provided a novel method for demonstrating the established theory among chemists that similar compounds have similar properties (23). Fig. 1A reveals that the compounds in THSWD possess a broad diversity in chemical space, which indicates that THSWD, as a natural combinatorial chemical library, has the potential to exhibit the desired interactions with a wider range of protein targets than the drug/drug-like compounds from the TTD. The marked overlap in Fig. 1B suggests that THSWD is likely to contain active compounds that have the potential to exert synergistic therapeutic actions. The statistics of the drug-like properties listed in Table II show that the means of the molecular weight, the number of hydrogen bond donors, the number of hydrogen bond acceptors and AlogP are 321.59, 3.19, 5.97 and 1.71, respectively. According to Lipinski’s rule of five (24), these results suggest that the majority of the compounds in THSWD have desired drug-like properties, and are suitable for screening lead compounds.

Using virtual docking screening, we demonstrated that THSWD contains certain potential promiscuous and combination drugs (50% for each); therefore, multiple active compounds in THSWD may simultaneously target several proteins associated with OA. In order to enhance our understanding of the functions of THSWD, the property profile of the potential compound-target interactions was compared with that of the candidate compound-target interactions, established from the TTD. The cC-cT network was generated by six connected components, with the candidate compounds in the outer circle of each component demonstrating fewer interactions with the target proteins. The mean number of candidate targets per candidate compound was 1.3. However, the pC-pT network consisted of 53 nodes (38 potential compounds and 15 potential targets) and 75 edges. The mean number of potential targets per potential compound was 2.0. Among the 38 potential compounds, nine were capable of acting on more than two targets. These potential compounds included 6-hydroxykaempferol-7-O-glucoside, folic acid, neocarthamin, safflor yellow A, ferulic acid, eugeniin, (Z)-5-hydroxy-3-butylidene-phthalide, carthamidin and amygdalin. Previous studies have revealed that the majority of these compounds exhibit biological activity against OA (25–28). A maximum of seven targets were correlated with one potential compound. Notably, the pC-pT network contained only one component, while the cC-cT network contained six. This indicated that the anti-OA action of THSWD was a result of holistic combinations attacking different targets, rather than a precise attack on a single target. Therapeutic polypharmacology involves the theory of treating multigenic, complex diseases by targeting multiple targets with one or more drugs, in order to effectively reset the regulatory network processes that are altered in the disease state (29). Therefore, therapeutic polypharmacology may explain why THSWD is effective in the treatment of OA.

In order to further understand the therapeutic polypharmacology of THSWD, a T-D network was contructed to study the correlation between the diseases and targets. As demonstrated in Fig. 5, the majority of the diseases were classified as neoplasms and nervous system and cardiovascular diseases, in addition to musculoskeletal diseases. This suggests that THSWD is capable of exerting broad pharmacological effects. Amygdalin, for example, was indicated as a potential compound in THSWD. Amygdalin may treat OA by targeting heme oxygenase (HO)-1, an enzyme that has been associated with cardiovascular and musculoskeletal diseases, as well as neoplasms. In addition, it has been demonstrated that amygdalin has exhibited therapeutic potential in the prevention and treatment of atherosclerosis (30). Furthermore. amygdalin may significantly reduce plasma viscosity, prolong thromboplastin time and reduce fibrinogen levels, whilst exerting few effects on whole blood viscosity (31). Amygdalin has also been used for the treatment of asthma, tumors, bronchitis, emphysema, leprosy and diabetes (32). Clinical observations have indicated the efficacy of THSWD in internal medicine, gynecology, andriatrics, dermatology and ophthalmology (33). Therefore, taking into account the multi-component and multi-target patterns of THSWD, it is possible that THSWD may improve the efficiency of treatments for other diseases, aside from OA. It would be useful to determine the potential pharmacological effects of THSWD, in order to aid the development of new and effective therapeutic drugs from THSWD.

In conclusion, we have constructed a novel modeling system, by integrating chemical space, virtual screening and network pharmacology, to investigate the molecular mechanism of action of THSWD, with regard to its clinical efficacy against OA. Our results demonstrated that THSWD has an improved structural diversity, compared with the selected drug/drug-like compounds from the TTD, and contains several active compounds capable of targeting multiple OA-related proteins. Furthermore, the cC-cT and pC-pT networks revealed that THSWD possesses the properties of promiscuous and combination drugs for multi-target OA therapies. Moreover, the T-D network indicates that THSWD had therapeutic potential against diseases other than OA, such as cardiovascular diseases and neoplasms. This suggests that distinct diseases may be treated with the same TCM formula. As a whole, these results indicate that TCM formulations contain compounds with multi-target capabilities and broad pharmacological effects.

## Figures and Tables

**Table I.** Fifteen key protein targets associated with osteoarthritis.

Protein	PDB code
ADAMTS-4	2RJP
TNF-α	2AZ5
iNOS	2Y37
COX-1	3NT1
COX-2	3N8X
MMP-1	966C
MMP-3	1C3I
MMP-8	1ZS0
MMP-9	1GKC
MMP-12	3RTS
MMP-13	3I7I
VDR	1DB1
PPARγ	2VSR
CDK2	3PXY
HO-1	3TGM

PDB, Protein Data Bank; ADAMTS-4, aggrecanase-1; TNF-α, tumor necrosis factor-α; iNOS, inducible nitric oxide synthase; COX, cyclooxygenase; MMP, matrix metalloproteinase; VDR, vitamin D nuclear receptor; PPARγ, peroxisome proliferator activated receptor-γ, CDK2, cyclin-dependent kinase-2; HO-1, heme oxygenase.

**Table II.** Mean, standard deviation, minimum and maximum values for the key variables of the compounds in Taohong Siwu decoction.

Variable	Mean	SD	Minimum	Maximum
Molecular weight	321.59	180.74	59.11	938.66
No. of hydrogen acceptors	5.97	5.64	0.00	26.00
No. of hydrogen donors	3.19	3.60	0.00	17.00
ALogP	1.71	3.62	−9.55	13.60
Molecular volume	219.39	111.99	53.16	538.85
Wiener index	2179.23	3322.42	9.00	18291.00
Zagreb index	115.26	71.47	12.00	368.00

SD, standard deviation.

**Table III.** Simple parameters of the (cC-cT) and (pC-pT) networks.

Simple parameters	pC-pT network	cC-cT network
Connected components	1	6
Network density	0.054	0.010
Network heterogeneity	0.636	2.976
Network centralization	0.083	0.382
Characteristic path length	4.576	3.800
Average no. neighbors	2.830	2.488
Shortest paths	2756 (100%)	47636 (71%)

pC-pT, potential compound-potential target; cC-cT, candidate compound-candidate target.

**Table IV.** Sixty-nine diseases correlated with the 15 potential target proteins.

Index	Disease
D1	Abdominal aortic aneurysm
D2	Acute lymphoblastic leukemia (ALL)
D3	Acute myeloid leukemia (AML)
D4	Adenomatous polyposis
D5	ACTH-secreting pituitary tumors
D6	Advanced lung cancer
D7	Advanced solid tumors
D8	Alzheimer’s disease
D9	Arthritis
D10	Asthma
D11	Atherosclerosis
D12	Autoimmune diseases
D13	B-cell malignancies
D14	Behcet’s disease
D15	Bladder cancer
D16	Brain cancer
D17	Breast cancer
D18	Carcinoma *in situ*, unspecified
D19	Carpal tunnel syndrome
D20	Chondrosarcoma
D21	Chronic lymphocytic leukemia (CLL)
D22	Chronic myeloid leukemia
D23	Colorectal cancer
D24	Crohn’s disease
D25	Diabetes mellitus
D26	Dysmenorrhea, unspecified
D27	Emphysema
D28	Endometriosis
D29	Gastro-intestinal ulcers
D30	Genitourinary tumors
D31	Gestational hypertension
D32	Guillain-Barré syndrome
D33	Heart failure
D34	Hepatocellular carcinoma
D35	Hormone-refractory prostate cancer
D36	Hyperimmunoglobulinemia D
D37	Inflammation
D38	Inflammatory bowel disease
D39	Insulin resistance
D40	Ischemia reperfusion injuries
D41	Ischemic heart disease
D42	Kaposi’s sarcoma
D43	Lung cancer
D44	Meningioma
D45	Multiple sclerosis
D46	Myocardial infarction
D47	Nasopharyngeal cancer (NPC)
D48	Neonatal hyperbilirubinemia, jaundice
D49	Non-Hodgkin’s lymphoma
D50	Noninsulin-dependent diabetes mellitus
D51	Non-small cell lung cancer
D52	Obesity
D53	Osteoarthritis
D54	Osteoporosis, unspecified
D55	Ovarian cancer
D56	Pain, unspecified
D57	Pancreatic cancer
D58	Pathological angiogenesis
D59	Peutz-Jeghers syndrome
D60	Prostate cancer
D61	Psoriasis
D62	Renal cell carcinoma
D63	Rheumatic diseases
D64	Rheumatoid arthritis, unspecified
D65	Squamous cell carcinoma
D66	Stroke
D67	Testicular cancer
D68	Thyroid follicular carcinoma
D69	Ulcerative colitis

ACTH, Adrenocorticotrophic hormone.

## Abbreviations:

THSWD: Taohong Siwu decoction;
TCM: traditional Chinese medicine;
ADAMTS-4: aggrecanase-1;
MMP: matrix metalloproteinase;
TNF-α: tumor necrosis factor-α;
iNOS: inducible nitric oxide synthase;
COX: cyclooxygenase;
CDK: cyclin-dependent kinase;
HO: heme oxygenase;
PPARγ: peroxisome proliferator-activated receptor-γ;
VDR: vitamin D nuclear receptor;
TTD: therapeutic target database
